# A spine segmentation method based on scene aware fusion network

**DOI:** 10.1186/s12868-023-00818-z

**Published:** 2023-09-14

**Authors:** Elzat Elham Yilizati-Yilihamu, Jintao Yang, Zimeng Yang, Feihao Rong, Shiqing Feng

**Affiliations:** 1 Department of Orthopaedics, Qilu Hospital of Shandong University, Shandong University, Jinan, China; 2 Jiangsu Shiyu Intelligent Medical Technology Co., Nanjing, China

**Keywords:** Spine, MRI, 3D segmentation, Deep learning

## Abstract

**Background:**

Intervertebral disc herniation, degenerative lumbar spinal stenosis, and other lumbar spine diseases can occur across most age groups. MRI examination is the most commonly used detection method for lumbar spine lesions with its good soft tissue image resolution. However, the diagnosis accuracy is highly dependent on the experience of the diagnostician, leading to subjective errors caused by diagnosticians or differences in diagnostic criteria for multi-center studies in different hospitals, and inefficient diagnosis. These factors necessitate the standardized interpretation and automated classification of lumbar spine MRI to achieve objective consistency. In this research, a deep learning network based on SAFNet is proposed to solve the above challenges.

**Methods:**

In this research, low-level features, mid-level features, and high-level features of spine MRI are extracted. ASPP is used to process the high-level features. The multi-scale feature fusion method is used to increase the scene perception ability of the low-level features and mid-level features. The high-level features are further processed using global adaptive pooling and Sigmoid function to obtain new high-level features. The processed high-level features are then point-multiplied with the mid-level features and low-level features to obtain new high-level features. The new high-level features, low-level features, and mid-level features are all sampled to the same size and concatenated in the channel dimension to output the final result.

**Results:**

The DSC of SAFNet for segmenting 17 vertebral structures among 5 folds are 79.46 ± 4.63%, 78.82 ± 7.97%, 81.32 ± 3.45%, 80.56 ± 5.47%, and 80.83 ± 3.48%, with an average DSC of 80.32 ± 5.00%. The average DSC was 80.32 ± 5.00%. Compared to existing methods, our SAFNet provides better segmentation results and has important implications for the diagnosis of spinal and lumbar diseases.

**Conclusions:**

This research proposes SAFNet, a highly accurate and robust spine segmentation deep learning network capable of providing effective anatomical segmentation for diagnostic purposes. The results demonstrate the effectiveness of the proposed method and its potential for improving radiological diagnosis accuracy.

## Background

The spine is a crucial part of the musculoskeletal system supporting the body and organ structures, and facilitating human activity and load transfer. It also serves as a protective barrier for the spinal cord that guards against mechanical shock such as impact. MRI (magnetic resonance imaging) [1] is the most widely utilized diagnostic tool for detecting spinal injuries or degenerative diseases in spine surgery [2]. Recent advancements in deep learning have greatly improved the accuracy of spine positioning, segmentation, and recognition in MRI. These developments have played a pivotal role in diagnosing and treating a variety of spinal conditions, including surgical planning, prognosis assessment, and image-guided intervention procedures [3]. However, due to the unique characteristics of MRI acquisition, neighboring vertebrae and different categories of vertebrae (intervertebral discs) can appear similar in shape and appearance, particularly in the first or last sagittal slices, making differentiation challenging. Furthermore, visual differences such as variations in illumination or contrast can further complicate the identification of intra-class vertebrae. Additionally, unlike 2D images which only have width and height dimensions, the depth dimension in 3D MRI increases the computational cost of the model.

Machine learning [4] techniques are widely utilized to extract essential information from MRI, such as vertebral bodies, spinal shapes, and intervertebral discs. In fact, locating anatomical structures in MRI datasets is often the primary objective for identifying and classifying pathological features or predicting prognosis. Peng [5] proposed a novel search approach that utilizes polynomial functions to fit the intensity distribution of all disc clues in a slice. Schmidt [6] introduced an efficient method for localizing anatomical structures based on parts, which incorporates contextual shape knowledge in a probabilistic graphical model. This method can even perform stable testing in cases where spinal images are obstructed. Oktay [7] developed a method for locating and labeling lumbar vertebrae and intervertebral discs in sagittal MRI slices with missing or abnormal structures, employing a Markov chain graphical model of ordered intervertebral discs and vertebrae in the lumbar spine, along with local image features and semi-global geometric information, to perform proportionally invariant localization of both intervertebral discs and vertebrae. Glocker [8] proposed an algorithm for typical feature localization and recognition of spinal pathology and image artifacts based on a supervised classification forest and avoids explicit appearance parameter models. However, in recent years, with the outstanding performance of artificial neural networks and deep learning in research, deep learning is increasingly adopted to locate spinal structures. Chen [9] proposed an innovative method for automatic vertebral recognition with the joint convolutional neural network (J-CNN) in 3D CT volumes. This cutting-edge model is capable of eliminating the detection errors of a set of rough vertebral centroids generated by a random forest classifier. On the other hand, Payer [10] utilized a regression technique that relies on the heat map of the target location to achieve localization in the variant anatomy space, which depends on a spatial network of precise local appearance responses and modeling of anatomical variation landmarks. In image analysis, understanding the content of an image is crucial, which involves segmenting an image into multiple regions at a pixel level so that each pixel belongs to a specific region. This process is known as semantic segmentation. In medical imaging, segmentation algorithms should not only identify whether a pixel belongs to the intervertebral disc, but also determine which instance a part of the segmentation belongs to. This type of segmentation is commonly referred to as an instance segmentation algorithm [11]. To evaluate the quality of segmentation algorithms, it is necessary to establish quantitative measures, with the most commonly used being the Dice similarity coefficient (DSC) and the mean surface distance (MSD). The DSC measures the spatial overlap between the segmentation image and the grand truth, while the MSD describes the average distance between each surface voxel of the segmentation surface and the closest surface voxel in the grand truth. Çiçek [12] proposed a volume segmentation algorithm, 3D Unet, which learns from sparsely annotated volume images. This algorithm utilizes a weighted loss function and targeted data augmentation, allowing 3D Unet to generate highly generalized results with minimal training data. Xiao [13] developed a new network, 3D ResUnet, by integrating Resnet, attention, and Unet and replacing each sub-module of Unet with a residual connection. This network model has demonstrated excellent performance on images with insufficient light sources. Zhou [25] proposed a method for rethinking semantic segmentation. Traditional semantic segmentation methods treat Softmax weights or query vectors as learnable class prototypes. However, this research reveals the limitations of such methods and presents a non-parametric alternative. The model uses a set of non-learnable prototypes to represent each class and relies only on the average features of a small number of training pixels. By employing a non-parametric nearest prototype retrieval approach, dense prediction is achieved. Chen [14] has extended DeepLabv3 by combining the spatial pyramid pooling module and encoder-decoder structure characteristics and adding a decoder module, thus forming a new network, DeepLabv3 + . The new DeepLabv3 + optimizes boundary segmentation, especially along the object's boundary, and further explores the Xception model by applying depth separable convolution to the Atrous spatial pyramid pooling and decoder modules, forming a faster and more powerful encoder-decoder network. Zhang [15] proposed the Cascade Fusion Network (CFNet) to enhance dense prediction performance. The main structure of this network is to insert feature operations into the backbone network, allowing more parameters for feature fusion and greatly increasing the richness of feature fusion. CFNet has surpassed ConvNeXt and Swin Transformer by 1% ~ 2% accuracy in object detection and instance segmentation tasks. Zhou [24] proposed a three-dimensional memory network named VMN for interactive segmentation of 3D medical images. This method utilizes a 2D interaction network to generate initial 2D segmentation for the selected slices and further refines it using an enhanced memory network and a quality assessment module.

For the segmentation model of spinal MRI, it faces the challenge of inter-class similarity and intra-class variation. Inter-class similarity refers to the high similarity between the first the last sagittal plane of the intervertebral discs (IVDs) in each sample, while intra-class variation refers to the visual differences among IVDs of the same category from different samples. To address these issues, we propose a segmentation method called Scene-Aware Fusion Network (SAFNet) that simultaneously segments the vertebral bodies and IVDs. The study extracts low-level, mid-level, and high-level features from the MRI and utilizes the correlation between different spinal structures to overcome the challenges of inter-class similarity and intra-class variation.

## Methods

**Fig. 1:**
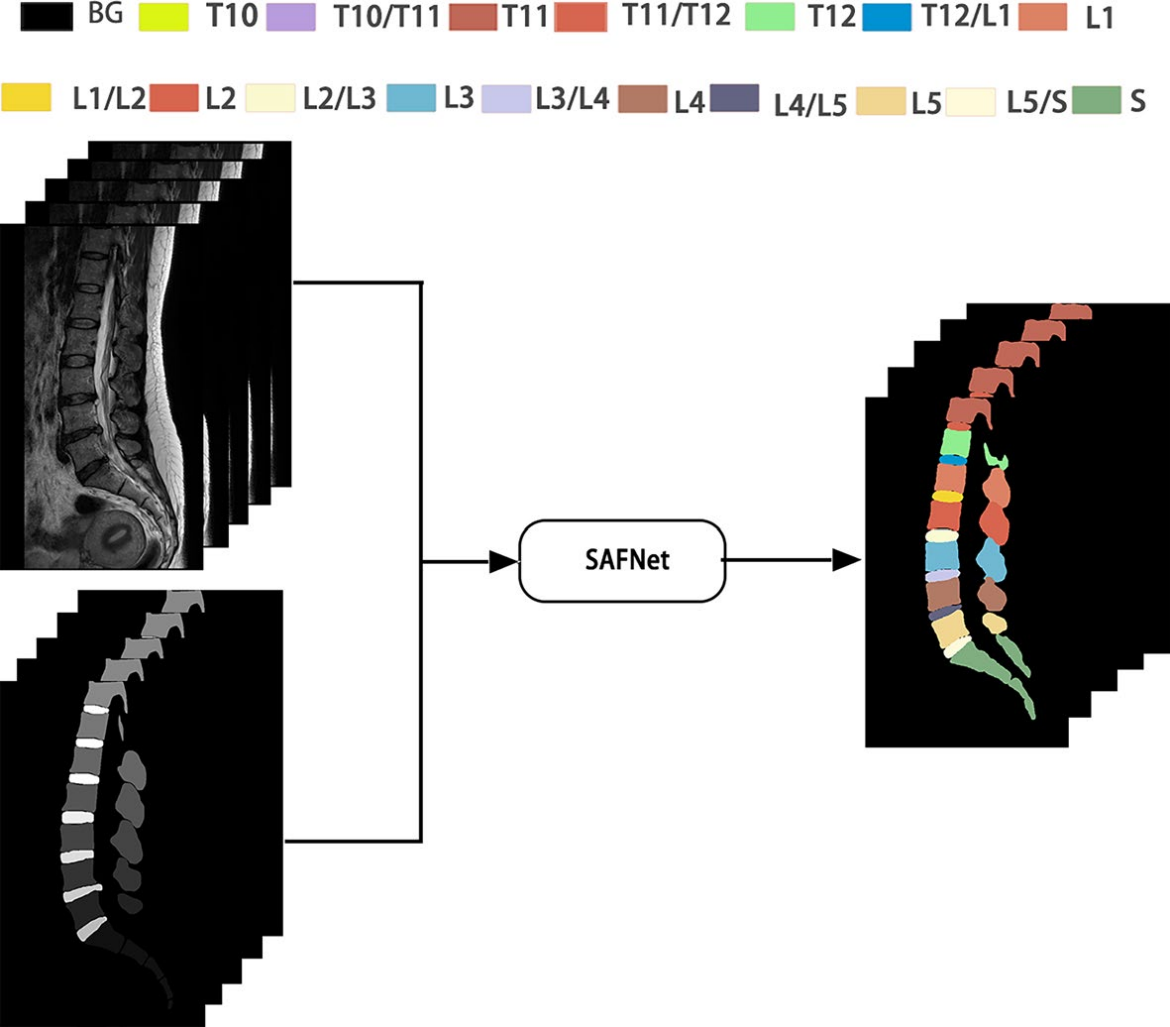
Spine parsing results. Spine parsing refers to the multi-class segmentation of both the vertebrae and intervertebral discs, whereby each individual vertebra or intervertebral disc is assigned its own unique label. The letters T, L, and S are used to represent thoracic, lumbar, and sacral vertebrae, respectively. (BG represents the background.)

To solve the issue of small inter-class differences and significant intra-class differences in spine MRI, along with the computational difficulties processing high-dimensional 3D images, a spin segmentation technique that utilizes a Scene-Aware Fusion Network (SAFNet) is proposed. The segment result is shown in Fig. 1. AFNet is composed of five modules: Feature extraction network, Atrous Spatial Pyramid Pooling, Self-attention mechanism, Multiscale fusion, and Dimension splicing.

### Feature extraction network

**Fig. 2:**
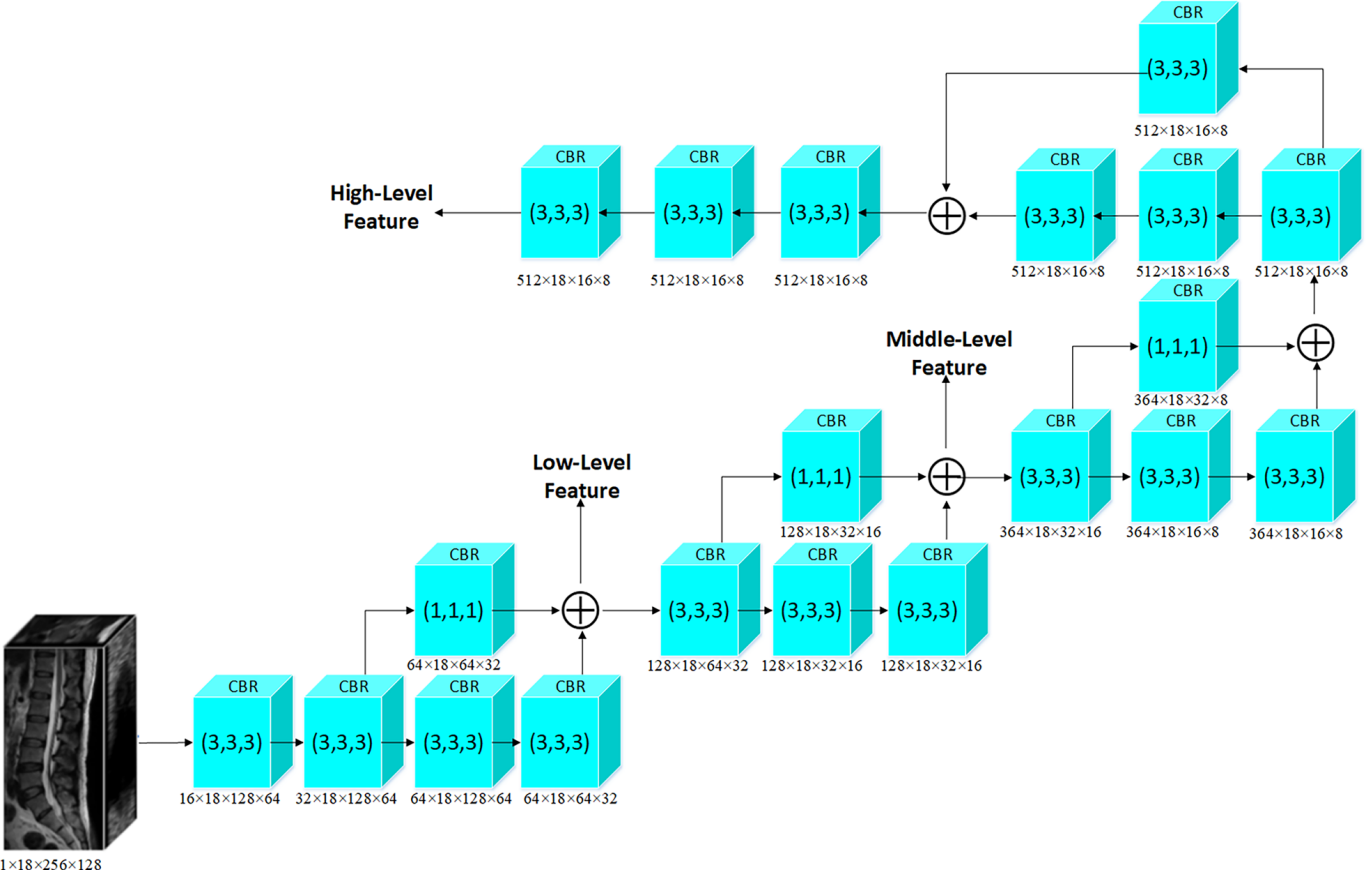
The structure of feature extraction network. The residual network structure uses cross-layer connections to directly pass input signals to subsequent layers and modify them in later layers to learn residual information. This network structure makes the training of SAFNet easier, while also improving its performance and convergence speed. In addition, residual structures can effectively reduce the number of model parameters and improve model generalization ability. (SAFNet denotes scene aware fusion network.)

SAFNet extracts low-level, mid-level, and high-level features from the input spinal magnetic resonance images by utilizing its structure as shown in Fig. 2. Before feature extraction, the input image undergoes CBR processing, which includes a 3D convolution with a kernel size of 3 × 3x3. The formula for CBR processing is presented below:

1 $$out\left({N}_{i},{C}_{outj}\right)=bias\left({C}_{outj}\right)+\sum_{k=0}^{{C}_{in}-1}weight({C}_{outj},k)\star input\left({N}_{i},k\right),$$

where $N$ represents the batch size, ${C}_{in}$ denotes the number of channels in the corresponding input image, $D$ represents the depth, $H$ represents the height, and $W$ represents the width of the image. $K$ denotes the kernel size and $\star$ signifies the valid 3D cross-correlation operator. The output undergoes normalization [16]:

2 $${B}_{out}=\frac{{B}_{in}-E[{B}_{in}]}{\sqrt{Var\left[{B}_{in}\right]+\epsilon }}\times \gamma +\beta ,$$

For each dimension over the mini-batches, the mean and standard deviation are calculated, $\gamma$ and $\beta$ are parameter vectors of size $C$ (where $C$ is the number of features or channels of the input) that can be learned. Afterward, the ReLu activation function is used to improve the nonlinearity of the feature map:

3 $$f\left(x\right)=\mathit{max}\left(0,x\right).$$

**Fig. 3:**
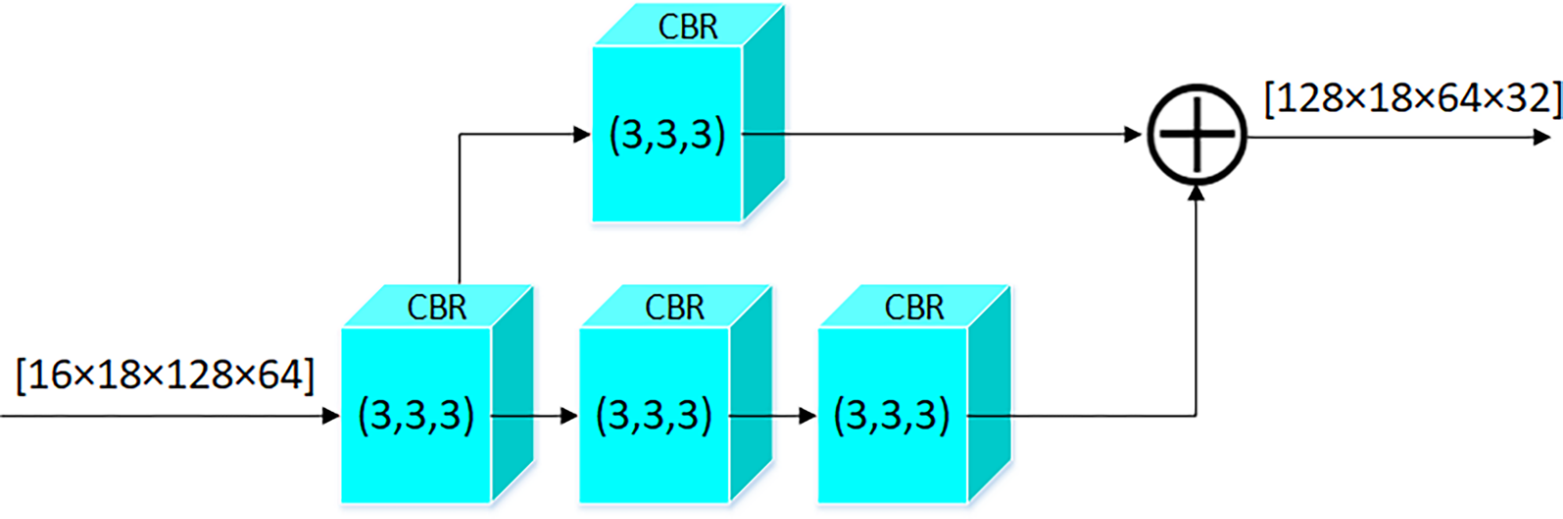
Residual network structure. The residual network structure uses cross-layer connections to directly pass input signals to subsequent layers and modify them in later layers to learn residual information. This network structure makes the training of SAFNet easier, while also improving its performance and convergence speed. In addition, residual network structure can effectively reduce the number of model parameters and improve model generalization ability. (SAFNet denotes scene aware fusion network; DSC denotes Dice Similarity Coefficient.)

Low-level features are extracted by applying a CBR process with a 3 × 3x3 kernel to the feature map that has been processed once. The stride is set to (1, 2, 2) causing the depth to remain the same while the width and height are halved. Next, a residual network is used to preserve the original information. The residual network structure is illustrated in the Fig. 3. A parallel branch is utilized for processing, where one branch applies a deeper CBR process using a 3 × 3 × 3 kernel and performs downsampling using a convolution with a stride of (1, 2, 2). The other branch processes the feature map using a CBR with a 1 × 1 × 1 kernel and stride of (1, 2, 2). The low-level features (128 × 18 × 64 × 32) are obtained by element-wise adding the results of the two branches. Subsequently, the low-level feature map is passed through another residual network to obtain mid-level features (128 × 18 × 32 × 16). Finally, the mid-level feature map is processed twice using the residual network process. The output image is then subjected to three CBR modules with a 3 × 3 × 3 kernel with holes to obtain high-level features (128 × 18 × 16 × 8).

The feature extraction network produces three sets of features with different sizes: low-level features (128 × 18 × 64 × 32), mid-level features (128 × 18 × 32 × 16), and high-level features (128 × 18 × 16 × 8). As the level goes higher, these features become increasingly rich in semantic information, while the detail information decreases due to a reduction in resolution. The focus of SAFNet is to fuse semantic information and detail information, and decode them to obtain the final spine segmentation result.

The feature extraction network extracts low-level features, mid-level features, and high-level features from the input MRI, is a commonly used approach in various previous researches. Li [26] proposed the Lesion-attention pyramid network for diabetic retinopathy grading (LAPN), where the feature extraction network has the ability to integrate images of different resolutions. Both the low-resolution and high-resolution networks are complete networks with own output branches. The output branch of the low-resolution network is used to obtain lesion activation maps, while the output branch of the high-resolution network is used for the final diagnosis. The entire network progressively fuses features and focuses on the features of the lesion area to achieve lesion-based diagnostic purposes.

In this paper, a convolutional and residual network was used to extract low-level, mid-level, and high-level features from the input images. CBR was employed to process the input images for feature extraction. The feature extraction network utilized residual networks to preserve the original information. In this network, the low-level features were obtained by applying CBR with different kernel sizes and strides, and added to the output of the residual network. Subsequently, the residual network was used again to extract mid-level and high-level features. The feature extraction network alleviated the problem of gradient vanishing by preserving and propagating the original information through the residual structure. Additionally, by fusing features extracted from different branches, the feature extraction network obtained richer semantic and detail information. This feature fusion enhanced the integrity and accuracy of the segmentation results.

### Atrous spatial pyramid pooling

To enhance the receptive field and capture multi-scale information, Atrous Spatial Pyramid Pooling (ASPP) [17] is utilized to process high-level features. ASPP is a spatial pyramid structure that employs dilated convolutions and has been widely applied in various iterations of Deeplab. Dilated convolutions [18] insert gaps between kernel elements during convolution, and the receptive field size is determined by the hyper-parameter (dilation rate). The formula for calculating the receptive field is as follows:

4 $$n=k+\left(k-1\right)\times \left(d-1\right),$$

where $d$ is the hyper-parameter of dilation. The size of the inserted space is $d-1$, while $k$ denotes the original convolution kernel size. The formula for calculating the size $o$ of the feature map after the hole convolution is as follows:

5 $$o=\left[\frac{i+2p-k-\left(k-1\right)\times \left(d-1\right)}{s}\right]+1.$$

**Fig. 4:**
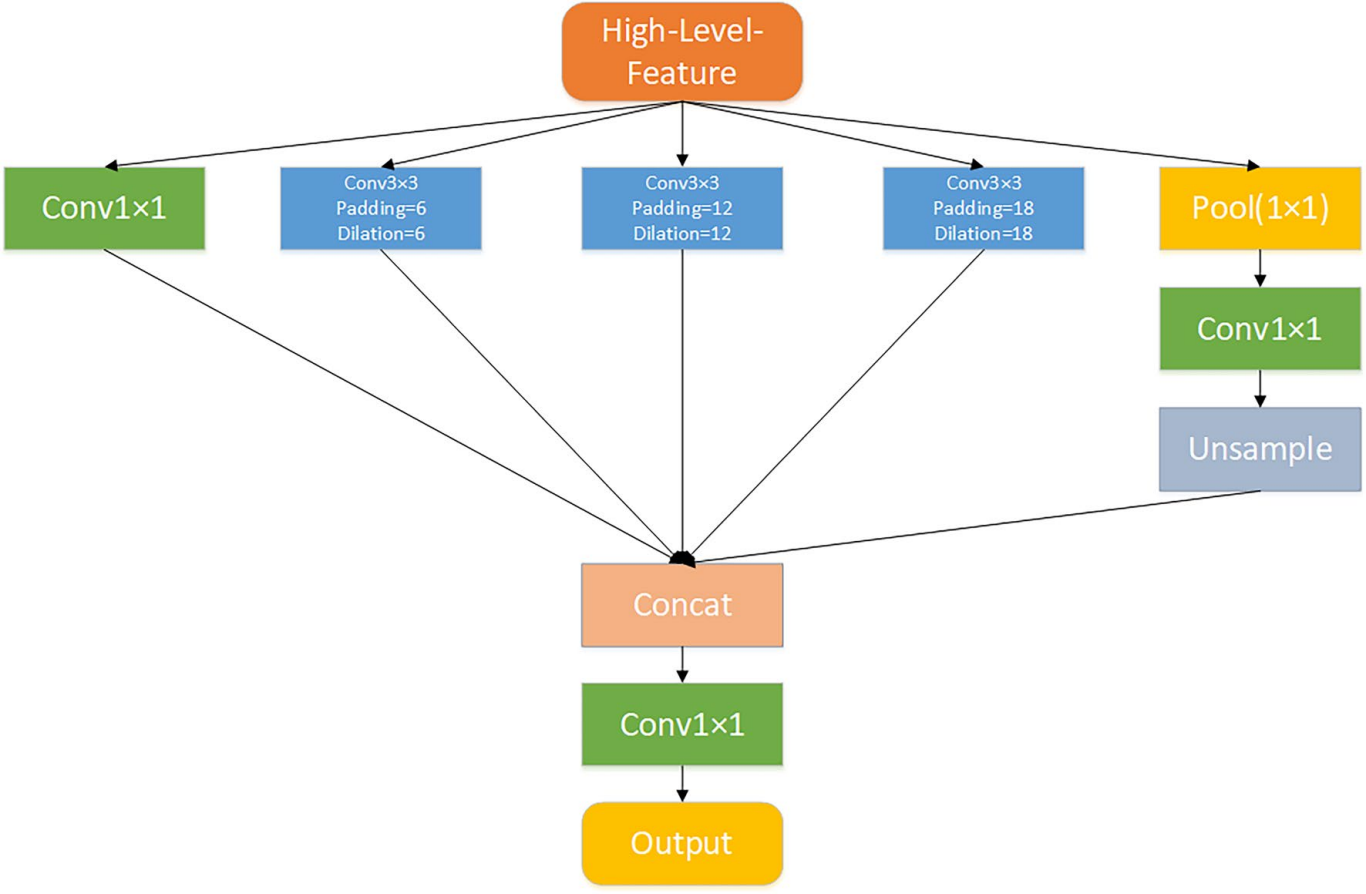
Atrous spatial pyramid pooling five-branch structure captures multi-scale information processing flow. Atrous Spatial Pyramid Pooling performs dilated convolutions with varying dilation rates on a single top feature map, and then concatenates the resulting feature maps to increase the number of channels. To achieve the desired number of channels, a convolution layer is used to reduce them. In this research project, a five-branch Atrous Spatial Pyramid Pooling structure was employed to process high-level features and broaden their receptive field for capturing multi-scale information

The dilated convolution takes an input size of $i$ and a stride of $s$, and its purpose is to increase the receptive field without using pooling and downsampling operations. Which allows each output of the convolution to obtain a wider range of information. ASPP's primary operation is to perform dilated convolutions with varying dilation rates on the same top feature map. The resulting feature maps are concatenated together to increase the number of channels. Finally, a convolution layer is used to reduce the number of channels to the desired value. In this research, ASPP is utilized to process high-level features and expand their receptive field to capture multi-scale information using a five-branch structure, as illustrated in Fig. 4. The branch structure is comprised of:

- Branch 1: use a 1 × 1 convolution to reduce the dimensionality of the input.
- Branch 2: use a 3 × 3 convolution layer with padding of 6 and a dilation rate of 6 to convolve the input.
- Branch 3: use a 3 × 3 convolution layer with padding of 12 and dilation rate of 12 to convolve the input.
- Branch 4: use a 3 × 3 convolution layer with padding of 18 and dilation rate of 18 to convolve the input.
- Branch 5: use a pooling layer with the same size as the input to pool the input to 1 × 1, then use a 1 × 1 convolution to reduce the dimensionality, and finally unsampled back to the original input size. (Upsampling is known as image enlargement or image interpolation mainly aiming to enlarge the original image.)

Finally, concatenate the outputs of these five layers, reduce the dimensionality to the given channel number using a 1 × 1 convolution layer, and obtain the final output.

Subsequently, the high-level features undergo processing through a self-attention mechanism in both the spatial and channel dimensions. The features are sequentially processed using spatial and channel operations.

### Self-attention mechanism

The self-attention mechanism [19] comprises the Position Attention Module and Channel Attention Module. It is an attention mechanism extracted from the feature map itself. For convolution, the receptive field size is restricted by the size of the convolution kernel, which typically necessitates stacking multiple layers to focus on the entire feature map. The main advantage of self-attention is its global focus, which can capture the global spatial information of the feature map through simple queries and assignments.

**Fig. 5:**
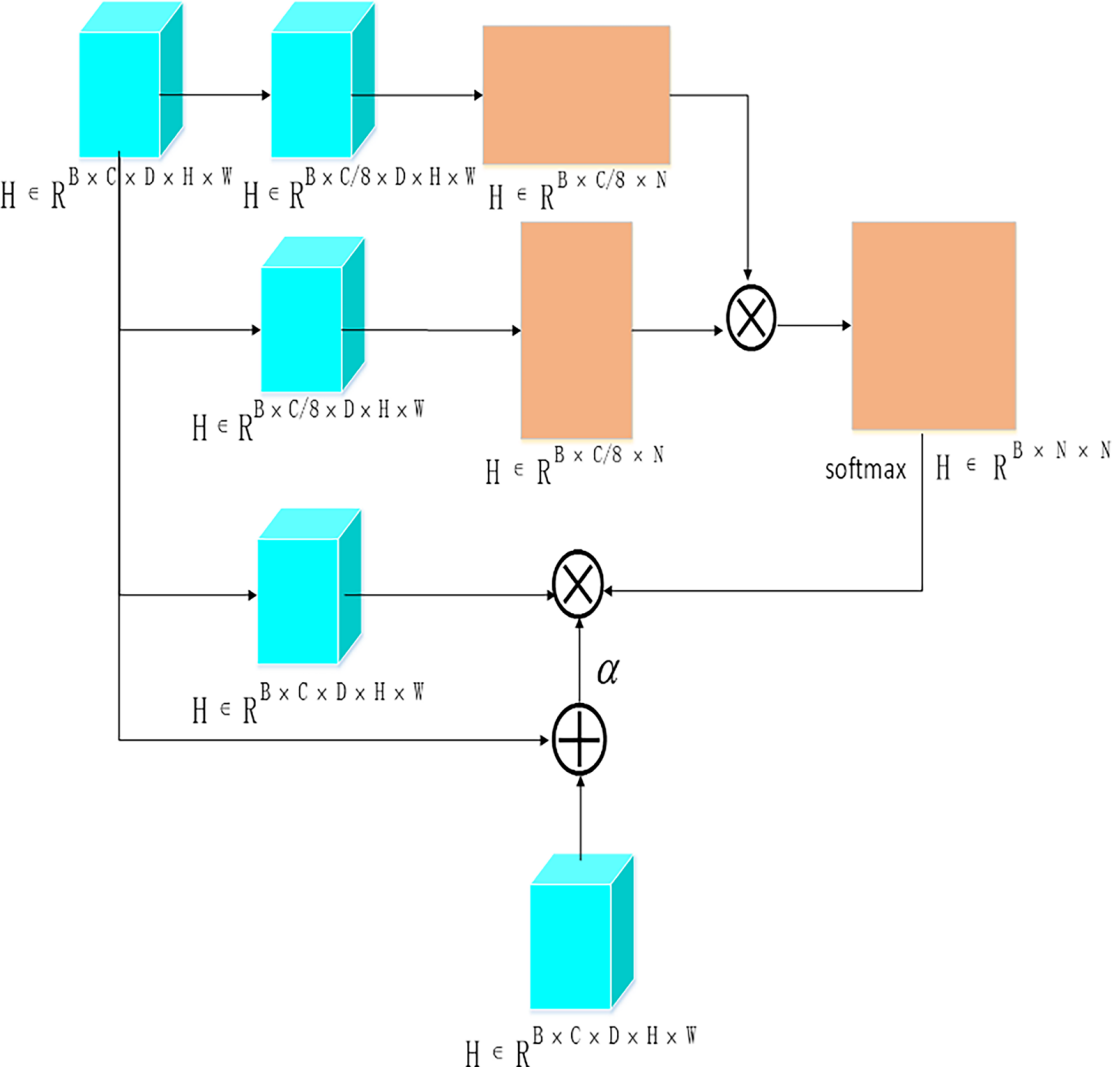
Position attention module. Position attention module effectively learns the dependency relationships between different positions, thereby improving the accuracy of feature representation. The Position Attention Module calculates the similarity between each position and other positions, and then weights them based on their similarity to aggregate information from different positions. Then, these weighted and aggregated feature information is sent to a fully connected layer for further reconstruction, which enhances the representation ability of features. Ultimately, the high-level features output by the Position Attention Module will contain more information about the relationship between different positions in the input features, which can help improve the model’s performance and generalization ability

In the Position Attention Module, as depicted in Fig. 5, the input feature ${\mathrm{R}}^{\mathrm{B}\times \mathrm{C}\times \mathrm{D}\times \mathrm{H}\times \mathrm{W}}$ is first subjected to a 3D convolution with a kernel size of (1, 1, 1) for dimensionality reduction. Following this, the spatial dimensions are flattened, which results in a feature of ${\mathrm{R}}^{\mathrm{B}\times \mathrm{C}/8\times \mathrm{N}}$, where N = D × H × W. Another parallel branch follows the same process and is then subjected to a matrix multiplication operation, which yields an N × N matrix. The matrix then undergoes a softmax operation to obtain the weight probabilities for spatial positions. This weighted matrix is then multiplied with the third branch, and the resulting output is connected to the input feature using a residual structure to obtain high-level features.

**Fig. 6:**
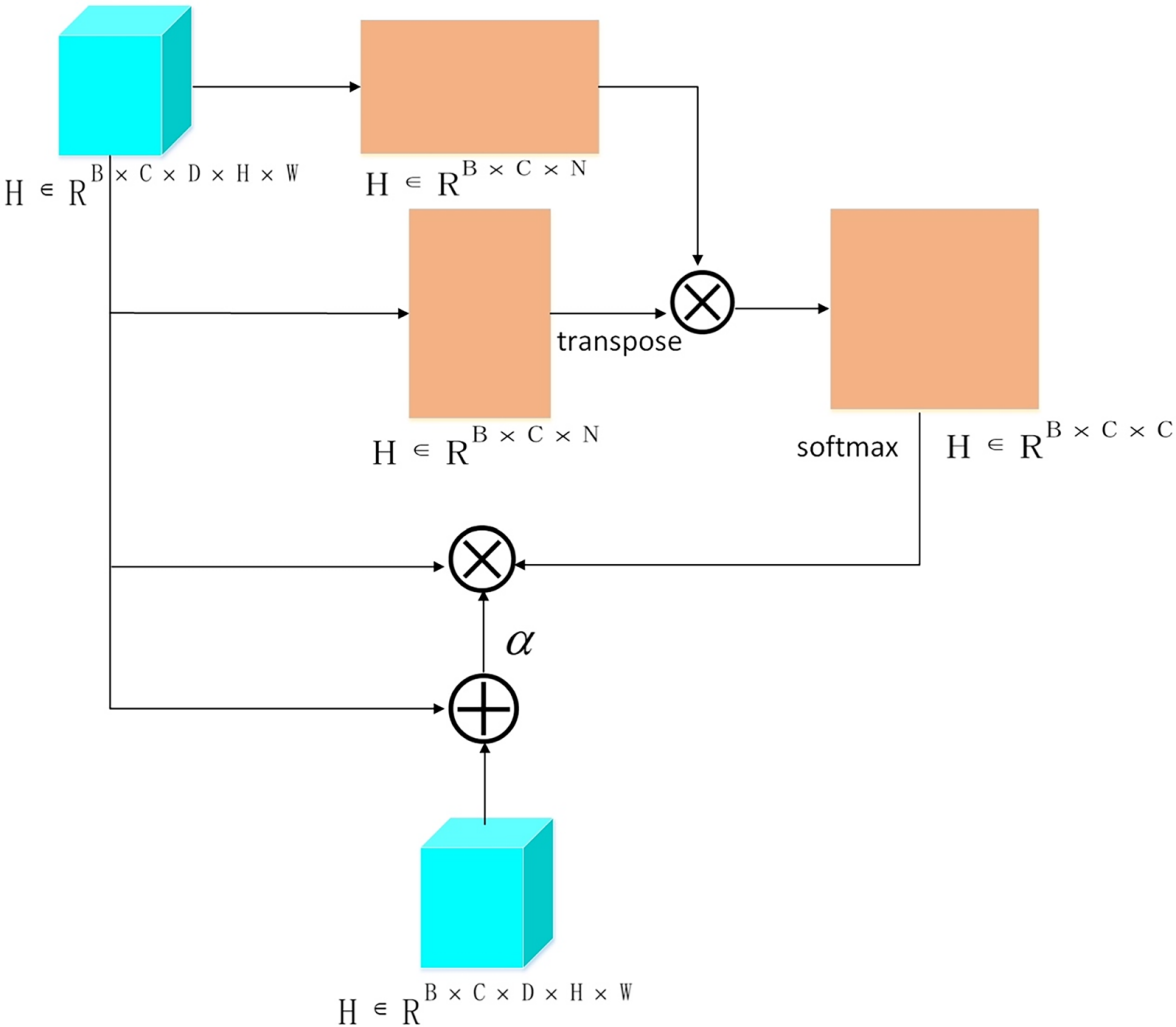
Channel attention module. Channel attention module weights and reconstructs different channels of input features to extract more accurate feature information. This module can learn the dependency relationships between each channel and perform adaptive adjustments on different tasks and datasets. Specifically, the Channel Attention Module calculates the importance of each channel and applies weighting across different channels so that the model can adaptively select the most useful feature information for decision-making. This process enhances the interpretability and generalization ability of the model and performs better in some complex tasks

Figure 6 illustrates that the high-level features ${\mathrm{R}}^{\mathrm{B}\times \mathrm{C}\times \mathrm{D}\times \mathrm{H}\times \mathrm{W}}$ obtained from the Position Attention Module are flattened to ${R}^{B\times C\times N}$, where N is equal to the product of D, H, and W. Another parallel branch undergoes the same processing and performs matrix multiplication, resulting in a C × C matrix. The matrix then undergoes a softmax operation to obtain the weight probabilities for spatial positions. This weighted matrix is then multiplied with the third branch, and the resulting output is connected to the input feature using a residual structure to obtain the high-level features processed by the Channel Attention Module.

The attention mechanism has been widely used in medicine by virtue of its capacity to automatically discover and focus on key features in images, accurately locate and identify abnormal or lesion areas in medical image tasks. Li [27] introduced a hybrid multi-head attention mechanism that can simultaneously focus on the correlations between different tasks and within individual tasks. This application of hybrid multi-head attention allows the model to better utilize the correlations between multiple tasks, improving generalization and effectiveness. Additionally, by incorporating spatial positional embedding, the model can better understand and utilize the correlations between different positions. This approach adds spatial information to the corresponding patches between tasks, which helps improve the representation capability of features. Furthermore, by integrating different attention heads, the model can synthesize different task-related information and generate more comprehensive and integrated feature representations. This approach enables the model to better capture the correlations between different tasks and improve the effectiveness of multi-task learning. Oktay [28] proposed a two-stage attention framework called Attention U-NET for medical image segmentation tasks. This method utilizes self-attention mechanism to capture the correlations of features at different levels, effectively controlling information flow and improving the accuracy and robustness of segmentation results. Attention U-NET can directly obtain global and local connections, and its results at each step are independent of the previous step, allowing parallel computation with fewer parameters and lower model complexity, which facilitates better model optimization. However, Attention U-NET has a fatal drawback of not being able to obtain positional information.

The attention mechanism used in this study is different from traditional attention mechanisms that only focus on the relationships between channels. It focuses on the spatial and channel correlations of the feature map through the position attention module and channel attention module, respectively. The advantage of this approach is that it comprehensively captures the information in the feature map and enhance the model's expressive power. Furthermore, residual structures are used in the position attention module and channel attention module. This structure helps to better transmit information between modules, alleviating the problem of gradient vanishing, and making the connections between modules tight, which facilitates effective feature propagation.

### Multiscale fusion

The low-level features possess a smaller receptive field and higher resolution while containing more positional and detail information. However, they tend to have lower semantic meaning and more noise since they have lower number of convolutions. On the other hand, the mid-level features have a stronger semantic information, but lower resolution and less perception of details. As a result, traditional object detection models often rely only on the last layer of the feature extraction network to classify and locate objects due to its high downsampling rate. This approach results in less effective information for smaller objects on the last feature map, which in turn reduces their detection ability. This problem is referred to as the multi-scale problem. To address this issue, researchers have explored the use of multi-scale fusion to efficiently integrate low-level and mid-level features [20]. The structure utilizes four parallel branches to process the input low-level or mid-level features to output new low-level and mid-level features. The specific structure is as follows:

- The first layer, dimensionality reduction (1/4), dilated ratio (1, 2).
- The second layer, dimensionality reduction (1/4), dilated ratio (1, 4).
- The third layer, dimensionality reduction (1/4), dilated ratio (1, 8).
- The fourth layer, dimensionality reduction (1/4), dilated ratio (1, 16).

Element-wise addition is performed on each branch, and the structures of the four branches are concatenated along the channel dimension. This enhances the scene perception ability of the low-level or mid-level features, while also preventing further reduction in resolution and increasing the receptive field.

### Dimension splicing

To obtain high-level feature map ${\mathrm{F}}_{\mathrm{h}}\in {\mathrm{R}}^{\mathrm{B}\times \mathrm{C}\times \mathrm{D}\times \mathrm{H}\times \mathrm{W}}$, middle-level feature map ${\mathrm{F}}_{m}\in {\mathrm{R}}^{\mathrm{B}\times \mathrm{C}\times \mathrm{D}\times \frac{\mathrm{H}}{2}\times \frac{\mathrm{W}}{2}}$, and low-level feature map ${\mathrm{F}}_{l}\in {\mathrm{R}}^{\mathrm{B}\times \mathrm{C}\times \mathrm{D}\times \frac{\mathrm{H}}{4}\times \frac{\mathrm{W}}{4}}$ using the self-attention mechanism, it is necessary to reduce the computational parameters and incorporate global information about the features. To achieve this, global adaptive pooling is utilized to obtain the channel response map. The formula for global adaptive pooling is as follows:

6 $${\mathrm{R}}_{GAP}=\mathrm{GAP}\left({\mathrm{F}}_{\mathrm{h}}\right),\mathrm{R}\in {\mathrm{R}}^{\mathrm{B}\times \mathrm{C}\times 1\times 1},$$

where ${\mathrm{R}}_{GAP}$ represents a new high-level feature, $GAP$ represents global adaptive pooling, and ${\mathrm{F}}_{\mathrm{h}}$ represents the high-level feature processed in self-attention mechanism. This operation averages the spatial dimensions of the feature maps to obtain a single channel response map that reflects the global information of the features. By performing global adaptive pooling, the computational parameters are reduced, and the self-attention mechanism can effectively capture global dependencies among features.

The new high-level features are activated using the sigmoid function, mapping features to the range of 0 to 1:

7 $${R}_{S}=Sigmoid\left({\mathrm{R}}_{GAP}\right),{\mathrm{R}}_{GAP}\in {\mathrm{R}}^{\mathrm{B}\times \mathrm{C}\times 1\times 1},$$

Using the attention mechanism module to process the activated ${R}_{S}$, and then multiplying the processed ${R}_{SAM}$ obtained by the attention mechanism module with ${F}_{l}$ and ${F}_{m}$ respectively, new high-level features are obtained:

8 $${F}_{sh}={R}_{SAM}\cdot {F}_{l}\cdot {F}_{m . }$$

The low-level features are represented by ${F}_{l}$, and the mid-level features are represented by ${F}_{m}$. The final high-level features ${F}_{sh}$ is sampled to have the same dimension as ${F}_{l}$ and ${F}_{m}$, and then concatenated along the channel dimension to produce the final result. This process ensures that the features across all scales are combined effectively and contribute to the final output. By incorporating features from multiple scales, the model can capture both local and global dependencies, yielding improved performance on complex tasks.

### Loss function

The research explores the use of pixel-wise cross-entropy loss to guide a 3D segmentation model in accurately classifying pixels in data. By minimizing this loss, the model can learn the ability to correctly classify each pixel, thereby achieving accurate 3D segmentation results:

9 $${L}_{pixelwise}=-\frac{\sum \sum \sum \sum [Y\left(i,j,k,c\right)\times log \left(P\left(i,j,k,c\right)\right)+(1-Y\left(i,j,k,c\right))\times \mathrm{log}(1-P\left(i,j,k,c\right))]}{H\times W\times D\times C}$$

where $H$ denotes height, $W$ denotes width, $D$ denotes depth and $C$ denotes number of categories. $Y\left(i,j,k,c\right)$ is the value of the number of categories of the $\left(i,j,k,c\right)$ pixels of the real label (0 or 1), $P\left(i,j,k,c\right)$ represents the predicted probability of the class for the $\left(i,j,k,c\right)$ pixel in the model's output.

### Dataset

The dataset used in this experiment is an open-source dataset from the second CSIG Image Graphics Technology Challenge. This research combined the original training and validation sets, with a total of 172 samples. Due to the limited number of samples, the model is prone to overfitting. To mitigate this, research employed cross-validation to evaluate the model and find the optimal configuration that resolves the overfitting issue. The core idea of cross-validation is to partition the dataset multiple times and take the average of the results from multiple evaluations to eliminate the adverse effects caused by unbalanced data division in a single split. Five-fold cross-validation can effectively reduce the variance of model evaluation results and improve the accuracy of model evaluation.

This research used a five-fold cross-validation method [21]. Specifically, we selected 138 samples as the training set, 4 samples as the validation set, and the remaining 30 samples as the test set. Since cross-validation involves random shuffling and combination, it can effectively increase the reliability of model performance evaluation. By utilizing cross-validation, we are able to evaluate the model's performance accurately and provide a robust assessment of its effectiveness on the dataset.

### Data preprocessing

The following steps are taken for all input images: cropping, resampling, padding, and normalization. To remove the parts of the MRI (${\mathrm{D}}_{0}\times {\mathrm{H}}_{0}\times {\mathrm{W}}_{0}$) without the spine, a bounding box of size ${\mathrm{D}}_{0}\times {\mathrm{H}}_{0}\times \frac{{\mathrm{W}}_{0}}{2}$ is used to crop the image around the center during the cropping stage. Next, the cropped MRI is resampled and padded to normalize the size to 18 × 256 × 128. Lastly, the MRI underwent normalization by subtracting the mean and dividing by the standard deviation.

### Comparison model selection

To assess the effectiveness of the proposed method, we selected four established spine segmentation methods for comparative experiments. These methods include CFNet [15], 3D DeepLabv3 [14], 3D ResUnet [13], and 3D UNet3D [12]. Detailed information and parameter design for each method are outlined below:

- Unet loss function is CrossEntroLoss, optimizer is Adam, and learning rate is ${10}^{-3}$.
- CFNet loss function is CrossEntroLoss, the optimizer is Adam, and the learning rate is ${10}^{-3}$.
- 3D DeepLabv3 loss function is CrossEntroLoss, the optimizer is Adam, and the learning rate is ${10}^{-3}$.
- ResUnet loss function is CrossEntroLoss, optimizer is Adam, and learning rate is ${10}^{-3}$.

### Training environment

The experiments are carried out using PyTorch 1.8.1 and CUDA 11.1 frameworks on two NVIDIA GeForce RTX 4090, each with 24 GB memory. The Adam optimizer is employed during experiment, with an initial learning rate of ${10}^{-3}$. The model underwent training for 50 epochs with batch size 8 and weight decay is ${10}^{-4}$. To adjust the learning rate dynamically, the ReduceLROnPlateau approach was employed as the learning rate scheduler. The mode was set to maximize accuracy. If there was no improvement in the validation accuracy for 10 consecutive rounds (patience = 10), the learning rate was reduced by a factor of 0.5. The data augmentation techniques used were random rotation, random contrast adjustment, and elastic deformation. The model saving process involved validating the model on the validation set after each epoch and retaining the model with the highest accuracy on the validation set throughout the training process.

## Results

**Fig. 7:**
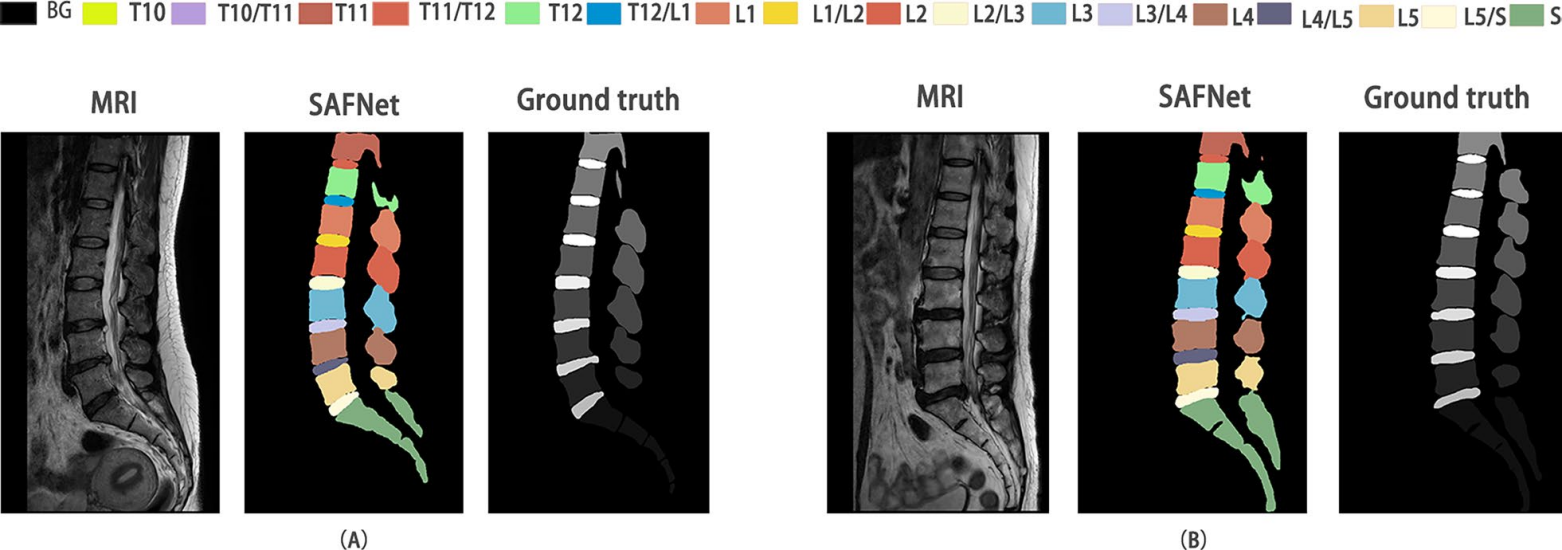
SAFNet segmentation MRI vertebral results. **A** and **B** are the results of the spinal mid-sagittal plane segmentation for six subjects using SAFNet. (BG represents the background; MRI denotes magnetic resonance imaging; SAFNet denotes scene aware fusion network; DSC denotes Dice Similarity Coefficient.)

As shown in Fig. 7, the proposed SAFNet achieved accurate vertebral segmentation in MRI. Original MRI, SAFNet segmentation and manually drawn segmentation for each group are shown. The SAFNet segmentation highly agrees with the manually drawn segmentation, especially in the S region in Fig. 7A, where the automatic segmentation can compensate for the area not drawn manually. This result is also reflected in Fig. 7A T12, where the manually drawn result did not depict the entire left side of T12, but the SAFNet model trained was able to well segment the area that is insufficiently segmented manually. These results indicate that the automatically segmented mask has highly overlaps with the manually drawn mask, and that SAFNet can achieve a balance between over-segmentation and under-segmentation.

**Fig. 8:**
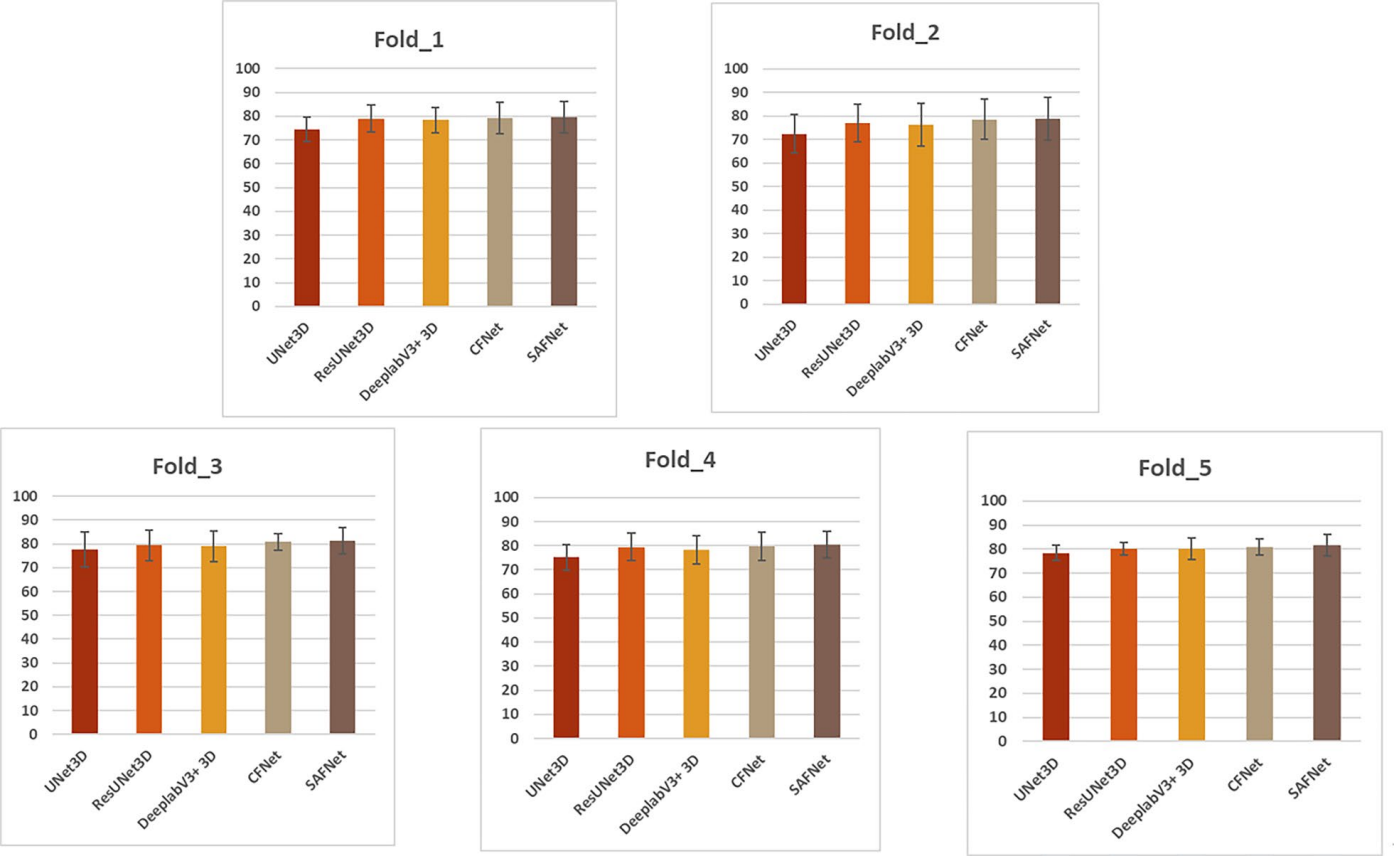
The mean DSC (%) for the most of individual vertebra segmentation visualization results. DSC is a commonly used indicator to evaluate the accuracy of segmentation, which can reflect the similarity between the model output and the ground-truth annotation results. SAFNet performs well in terms of vertebral segmentation visualization effect, can effectively extract and represent vertebral structure information, and achieves the highest average DSC for most single vertebral segmentation visualization results on multiple testing datasets, indicating that the model has high stability and generalization ability. (SAFNet denotes scene aware fusion network; DSC denotes Dice Similarity Coefficient.)

Table 1 and Fig. 8 show the mean DSC results of SAFNet and four other comparative models for the segmentation of 17 spinal structures in 5 folds. In all 5 folds, the averages DSC of SAFNet are 79.46 ± 4.63%, 78.82 ± 7.97%, 81.32 ± 3.45%, 80.56 ± 5.47%, and 80.83 ± 3.48%, with a mean DSC of 80.32 ± 5.00%. These results indicate that SAFNet exhibited high stability in the 5 folds. Specifically, the mean DSC values of SAFNet showed high consistency and stability in each fold. For all 17 spinal structures, SAFNet also performed the best, indicating that the model is excellent in accuracy and reliability in spinal segmentation. These results provide strong support for the practical application of SAFNet.

**Table 1:** SAFNet Achieves the Highest Mean DSC (%) for Most Individual Vertebra Segmentation

Baseline	Fold_1 (%)	Fold_2 (%)	Fold_3 (%)	Fold_4 (%)	Fold_5 (%)
3D UNet	74.42 ± 5.22	72.45 ± 8.24	75.68 ± 7.42	74.22 ± 5.32	78.35 ± 3.22
CFNet	78.95 ± 5.61	77.02 ± 8.14	78.31 ± 6.46	74.46 ± 5.67	80.13 ± 2.64
3D ResUNet	78.95 ± 5.41	76.27 ± 9.07	79.04 ± 6.38	75.25 ± 5.76	80.07 ± 4.49
3D DeepLabV3	79.19 ± 6.52	78.62 ± 8.48	82.77 ± 3.46	72.59 ± 5.88	80.83 ± 3.48
SAFNet	79.46 ± 4.63	78.82 ± 7.97	81.32 ± 3.45	80.56 ± 5.47	81.45 ± 3.47

Based on the DSC results, SAFNet outperformed 3D DeepLabV3 in all but Fold_3. In terms of standard deviation, SAFNet had the highest standard deviation in Fold_2, reaching 7.97%. However, the average standard deviation of all five models in Fold_2 reached 8.38%. Additionally, the average DSC value of the five models in Fold_2 (only 76.64%) is the lowest among the five training sets. The inter-class difference in the MRI in Fold_5 is the smallest, and its standard deviation (3.46%) is the lowest among the five training sets. Furthermore, the average DSC value of the five models in Fold_5 is the highest among the five training sets, reaching 80.12%. These findings suggest that the inter-class difference in the MRI in Fold_2 is too large to be suitable for model training, while the inter-class difference in the MRI in Fold_5 is the smallest. These conclusions also indirectly prove that the five-fold cross-validation has an excellent judgment effect on the rationality of dataset distribution. Moreover, the results of the five-fold cross-validation demonstrate that SAFNet is capable of achieving superior segmentation results under various different distribution datasets.

### Ablation experiments

In this research, SAFNet consists of five components, and we conducted a series of ablation experiments to evaluate the impact of different methods have on the results. We used mDice as the evaluation metric, and the results of the ablation experiments are shown in Table 2. First, we used the Feature extraction network model as the Base model. The mDice score of the Basic model was 77.15 ± 7.63%, which served as a baseline for subsequent experiments. Next, we introduced the ASPP (Atrous Spatial Pyramid Pooling) module into the Basic model. The experimental results demonstrated that the model using the ASPP module achieved an mDice score of 81.12 ± 5.20%, exhibiting significant improvement compared to the Base model. Then, we tried Multiscale fusion by applying it to the Base model. The experimental results showed that the Base model with Multiscale fusion achieved an mDice score of 80.53 ± 4.78%. Finally, we embedded the Self-attention mechanism method into the Base model. The results suggested that the model using the Self-attention mechanism method reached an mDice score of 79.76 ± 4.96%. Finally, we retrained SAFNet. The results showed that SAFNet achieved an mDice score of 81.37 ± 3.68% that further exceeds the other methods.

**Table 2:** Ablation experimental results for SAFNet

Methods	mDice (%)
Base	77.15 ± 7.63
Base + ASPP	81.12 ± 5.20
Base + Multiscale fusion	80.53 ± 4.78
Base + Self-attention mechanism	79.76 ± 4.96
(Ours)SAFNet	**81.37 ± 3.68**

In summary, the ablation experiments compared and validated the effectiveness of introducing the ASPP, Multiscale fusion, and Self-attention mechanism methods in improving segmentation accuracy, demonstrating the superiority of our proposed methods in this regard. These experimental results indicate that our method has significant advantages in segmentation performance for this task and can serve as a basis for further research and applications.

## Discussion

**Fig. 9:**
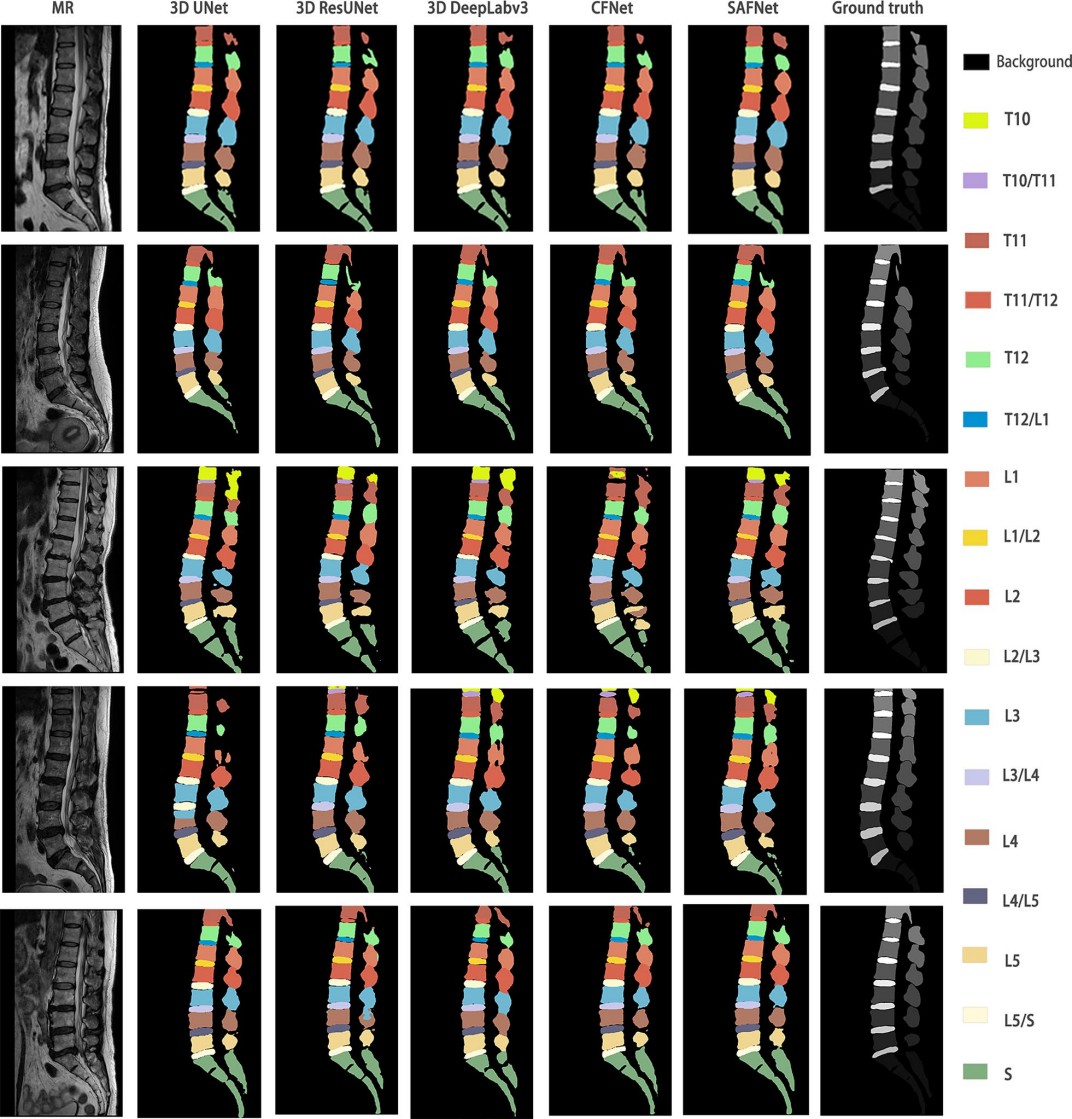
SAFNet has the ability to enhance the differentiation of each vertebra and intervertebral disc. SAFNet is designed to extract and learn semantic features from the input medical images, which can effectively distinguish different anatomical structures within the spine. Each row in the figure represents the middle sagittal slice of a subject. (SAFNet denotes scene aware fusion network; BG denotes background; MRI denote magnetic resonance imaging)

Five sets of images are randomly selected from the validation set and are presented in Fig. 9 for comparative purposes. It is noteworthy that SAF achieved the highest DSC in each image set. Images in the figure include the initial MRI, the segmentation results of 3D UNet, 3D ResUNet, 3D DeepLabv3, CFNet, SAFNet, and the ground truth.

In the first set of results, SAFNet accurately distinguished each vertebra structure, but due to its balance between over-segmentation and under-segmentation, it failed to fully reflect the convexity and concavity edges of the vertebral bodies in some areas, such as the edge of L5. Moreover, in the sacrum of the S section, SAFNet could not depict the intervertebral space of the sacral vertebrae like 3D DeepLabv3 or 3D ResUnet. In the second set of results, SAFNet reached a good balance between over-segmentation and under-segmentation, but still lagged behind 3D ResUnet in the depiction of some details. The third set of results is the worst among all MRI recognition results. ResUNet, UNet and CFNet produced confused category segmentation results, and all three segmentation networks failed to correctly segment T10 and T11. However, SAFNet had better overall segmentation outcomes, and 3D DeepLabv3 performed similarly to SAFNet. In the fourth set of results, UNet made recognition errors of T10, T10/T11, T11, and almost all right vertebral bodies. 3D ResUnet failed to recognize the T10 vertebra. 3D DeepLabv3 and CFNet had recognition defects on T12 and L1. SAFNet could correct the manual delineation errors of T10 and L4 but could not correctly complete the right sacral vertebrae. Although part of the outline was depicted, it was far from a complete outline. Finally, in the fifth set of results, ResUNet made classification recognition errors, while Unet and CFNet had serious missing problems in the recognition of T11. In comparison, SAFNet produced more favorable results on the T12 vertebra, while 3D DeepLabv3 did better in L4 and L3 recognition.

Through analyzing the automated and visual segmentation outcomes, it is apparent that SAFNet's performance in segmentation details and sacral vertebrae segmentation is inferior to that of 3D DeepLabv3 and ResUNet. However, SAFNet is the only model that did not make any errors in category segmentation, demonstrating high overall accuracy and stability. Despite SAFNet's inferior performance in certain details and sacral vertebrae segmentation compared to other models, its remarkable overall accuracy and stability in practical applications make it a promising vertebral segmentation model.

Doctors require extensive image reading experience to make a diagnosis in practical radiology examinations. The diagnosis of orthopedics relies not only on image segmentation but also on data such as vertebral body length, angle, and displacement distance [22]. Collecting this data usually requires identifying key points in the anatomical structure [23], which is completed through relevant mathematical calculations. However, certain deviations in each positioning may occur due to differences in doctors' cognition and other factors, making it difficult to establish uniform standards for doctors. In contrast, SAFNet has a unified standard and high stability, ensuring consistent segmentation and avoiding these problems.

Stability is a critical factor in evaluating the performance of spine segmentation models. A good segmentation model can effectively distinguish anatomical structures, and consequent accurate positioning can improve the accuracy of diagnosis data.

In view of the foregoing, SAFNet aims to pursue higher stability and overall accuracy, even though there is still room for improvement in some details of spine segmentation.

## Conclusion

This research proposes an accurate and stable deep learning SAFNet for spine analysis. SAFNet utilizes a scene-aware fusion network to address the challenges posed by small inter-class differences, large intra-class differences, and the computationally intensive nature of high-dimensional 3D spine MRI, while also improving segmentation accuracy. Results demonstrate the effectiveness of the proposed method and its potential for improving the accuracy of radiological diagnosis.

## Data Availability

The dataset used in this research is an open-source dataset from the 2nd China Society of Image and Graphics (CSIG) Image and Graphics Technology Challenge: MRSpineSeg Challenge: Automated Multi-class Segmentation of Spinal Structures on Volumetric MR Images (https://www.spinesegmentation-challenge.com) and are available from the corresponding author on reasonable request.

## Abbreviations

MRI: Magnetic resonance imaging
SAFNet: Scene aware fusion network
ASPP: Atrous spatial pyramid pooling
MSD: Mean surface distance
DSC: Dice similarity coefficient
